# Supplementary material for: Chromothripsis during telomere crisis is independent of NHEJ, and consistent with a replicative origin
Source: Genome Res. 2019 May;29(5):737–49. doi: 10.1101/gr.240705.118 (PMC6499312; doi:10.1101/gr.240705.118)
Supplement: Supplemental Material [file supp_gr.240705.118_Supplemental_file_1.zip › contigs/annotated_contigs/DB73/contig.2.DB73_length_296_mean_cov_9.12162162162.docx]

**DB73_length_296_mean_cov_9.12162162162**

```
GTGGTGGCGGGCGCCTGTAGTCCCAGCTACTCGGGAGGCTGAGGCAGGAGAATGGCGTGAACCCGA|ATATTATAACTTACA|CGGCAG
 >chr10:17462271-17462337 + E=2e-28                                               >chr3
ACCATGTGTGTATAGGAAAAATACAGAGTTGAGTTCAGCATAAAATAAGATTCTACAATCCTTCGAGCCTTTTCGCTGATGTCATAAAA
:189690676-189690891 + E=2e-118
CAGACGAGCGCTCTTCAGTGGGACTCGACCTTCATAACCAGACTGAAAAAAAAAAACAAAACATGAGAAACAAATGGGTTCATTGGAGG

TCGTAAGAAAAACAGGCACTAGCTTGCATTG
```
